# Optimal positioning of storage systems in microgrids based on complex networks centrality measures

**DOI:** 10.1038/s41598-018-35128-6

**Published:** 2018-11-09

**Authors:** Saman Korjani, Angelo Facchini, Mario Mureddu, Guido Caldarelli, Alfonso Damiano

**Affiliations:** 1 University of Cagliari, Department of Electrical Engineering, Cagliari, Italy; 2 IMT School for Advanced Studies, Lucca, Italy; 3 Italian National Research Council - Institute for Complex Systems, Rome, Italy

## Abstract

We propose a criterion based on complex networks centrality metrics to identify the optimal position of Energy Storage Systems in power networks. To this aim we study the relation between centrality metrics and voltage fluctuations in power grids in presence of high penetration of renewable energy sources and storage systems. For testing purposes we consider two prototypical IEEE networks and we compute the correlation between node centrality (namely Eigenvector, Closeness, Pagerank, Betweenness) and voltage fluctuations in presence of intermittent renewable energy generators and intermittent loads measured from domestic users. We show that the topological characteristics of the power networks are able to identify the optimal positioning of active and reactive power compensators (such as energy storage systems) used to reduce voltage fluctuations according to the common quality of service standards. Results show that, among the different metrics, eigenvector centrality shows a statistically significant exponential correlation with the reduction of voltage fluctuations. This finding confirms the technical know-how for which storage systems are heuristically positioned far from supply reactive nodes. This also represents an advantage both in terms of computational time, and in terms of planning of wide resilient networks, where a careful positioning of storage systems is needed, especially in a scenario of interconnected microgrids where intermittent distributed energy sources (such as wind or solar) are fully deployed.

## Introduction

In recent years energy production is experiencing a paradigm shift towards more clean and sustainable Renewable Energy Sources (RES). Among the emerging technologies, both wind and photovoltaic (PV) generators are considered the most promising energy sources of the future under technical and policy implementation point of view^1,2^. Indeed, RES offer clear advantages in terms of polluting emissions, energy availability, and easiness in the deployment of energy production facilities. Together with these advantages, this class of energy sources present significant drawbacks in terms of uncertainty of power production and intermittency, representing a challenge for the traditional concept of unidirectional power network based on large fossil-fueled power plants. Furthermore, such uncertainty may make worse power quality management, triggering instabilities in power systems both in terms of frequency and voltage control^3^. Moreover, the more the uncertainty, the more instabilities have been observed^4^, making the increasing penetration of RES the main source of quality of service (QoS) disturbances.

To overcome these issues, two complementary approaches have been proposed: the active control of loads and power flows, and the passive enhancement of the grid resilience^5^. By means of the first approach, power grids are experiencing huge technological advancements leading to a smart and real time management and control of their operative condition. The growing implementation of load control features in terms of smart meters, the possibility of nearly real time monitoring of the system operative parameters by means of SCADA systems, and the wide adoption of Energy Storage Systems (ESS) is enabling a fine grained control of these systems^6^. In particular, ESS are considered crucial for the full adoption of RES, because of their ability to limit network unbalances caused by fluctuating RES, by storing the surplus of energy produced for a future use. Furthermore, ESS are important under the economical point of view thanks for the increase system stability that they ensure, even considering limits like degradation due to calendar and cycle aging^7,8^ and the installation costs, that according to the type of use may hamper the lifetime of ESS reducing the Return of Investments^9^. On the other hand, increasing attention has been also paid in the definition of grid planning features oriented to the enhancement in terms of passive resilience, and, particularly in last years, the complex networks methods has been proven to be a solid framework for capturing and describing complex phenomena in power grids^4,10,11^. By example, complex networks have shown how the topology of power grids strongly impacts on the network robustness to attacks and failures^5,12,13^, to the grid synchronization^14^, to its overall voltage regulation^4^, and to its coordination with electric mobility energy needs^15^. This in turn showed how the correct planning of power grid topology can enhance the network overall resilience, decreasing in this way the costly implementation and maintenance of active control systems.

According to the literature, heuristic methods for finding the optimal location of the ESS in the networks with regards of voltage regulation, have been proposed by^7,16,17^, but such approaches are limited by the size and topology of the the network, leading to results referred to specific case studies, while there is still the need for a criterion able to cope with complex and wide topologies, with low computational effort, and more accuracy. In this sense, using a multi-disciplinary approach bridging complex networks science and electrical engineering appears to be promising.

Here we propose a general approach able to put in evidence how the centrality of the power network nodes can be used as a criterion for the optimal placement of ESS. We compute the impact of ESS on the network by means of a recent method based on genetic algorithms and optimal power flow^9^, further improved with a step for the reactive power optimization. Results show that there is a statistically significant correlation between the node eigenvector centrality and the optimal position of ESS, with a positive impacts on their voltage regulation abilities, and a overall reduction of the voltage fluctuations by a value up to 50%, significantly increasing the power quality.

## Results

The study has been performed on two prototypical IEEE 33 and 69 Bus medium voltage networks commonly used as a reference in technical literature^8,18–20^ (see methods for further details).

Considering an ESS supplying both active and reactive power, and starting from real generation and consumption data sampled every 60 minutes (for a total length of 720 samples, for a better description of the dataset the reader is referred to^8^), we have produced for each network 100 random configurations shuffling the loads and generators of each node of the network. This produces different temporal and spatial loads and at the same time keeps constant the total power produced and consumed by the network. The dimension of the ESS has been computed following^8^, and is kept constant in all the generated configurations. This step allows to cover as much as possible the possibility to find significant loads or significant generators in different nodes of the networks. The aim has been to guarantee the homogeneity of the possible power configurations both in the 33 and 69 bus cases, filtering in this way the impact of effects not strictly related with the network topology.

For each configuration we then compute the optimized impact of the ESS on the voltage stability of the system by means of Genetic Algorithm-based Multi-period Optimal Power Flow (GA-MPOPF) recently published^9^, and extended with a reactive power optimization step presented in the methods section. The effect of the ESS position has been performed by placing it in all the system nodes.

In this way, the optimal active and reactive power rate of the ESS has been calculated for each position. After this step, we calculated the set of observed voltages in the system during an entire month, per each of the possible positions of the ESS. Then, the amplitude of voltage fluctuations during the simulated month have been compared with different centrality measures (betweenness, eigenvector, closeness and pagerank) of the grid nodes.

To understand if batteries placed in the optimal position according to centrality metrics lead to positions that are optimal under the point of view of power flows, we studied the correlation properties between the computed nodes centrality and the interquartile difference Δ_*q*_ of the voltage fluctuation observed by placing the ESS in each node (see methods section for further details). The higher the correlation value, the higher is the accuracy of the optimal position found with the centrality driven criterion.

We start by computing the centrality metrics of the two networks. Table 1 shows the values of the Eigenvector centrality for the nodes of the 33 bus network for all the weighting models considered in this paper. As the reader can see, there is a set of nodes whose centrality is very low (e.g. nodes 25 to 33) and other nodes where centrality is several orders of magnitude higher (e.g. nodes 2, 3 23, 24).

**Table 1:** Values of the Eigenvector centrality for each node of the IEEE 33 bus network considering the different weights used to model the network.

Nodes	Eigenvector Centrality			
	1/|*R* + *jX*|	1/*R*	1/*X*	W/o
2	0.40681	0.39342	0.40679	0.10004
3	0.07698	0.07617	0.07697	0.11439
4	0.01786	0.022601	0.01786	0.08411
5	0.00130	0.010768	0.00134	0.07198
6	0.00025	0.001352	0.00026	0.07541
7	3.39E-05	0.000146	3.39E-05	0.04757
8	2.65E-05	6.90E-05	2.65E-05	0.03001
9	9.15E-06	1.63E-05	9.15E-06	0.01893
10	3.56E-07	9.99E-07	3.56E-07	0.01194
11	2.26E-08	1.66E-07	2.26E-08	0.00753
12	1.93E-09	2.50E-08	1.93E-09	0.00474
13	1.59E-10	2.95E-09	1.59E-10	0.00298
14	4.17E-12	2.05E-10	4.17E-12	0.00187
15	3.54E-13	3.46E-11	3.54E-13	0.00116
16	1.02E-13	1.86E-11	1.02E-13	0.00071
17	3.39E-15	1.14E-12	3.39E-15	0.00039
18	3.20E-16	2.44E-13	3.20E-16	0.00018
19	0.09799	0.095891	0.09798	0.06214
20	0.00478	0.012351	0.00478	0.03768
21	0.00071	0.002432	0.00069	0.02135
22	4.41E-05	0.000237	4.41E-05	0.00963
23	0.00490	0.008265	0.00491	0.06935
24	0.00038	0.000996	0.00038	0.03930
25	0.00016	0.000431	0.00016	0.01773
26	1.73E-05	0.000126	1.73E-05	0.04755
27	5.38E-06	3.91E-05	5.38E-06	0.02997
28	2.59E-07	3.29E-06	2.59E-07	0.01886
29	1.71E-08	3.70E-07	1.71E-08	0.01182
30	2.97E-09	6.46E-08	2.97E-09	0.00734
31	1.40E-10	6.37E-09	1.40E-10	0.00445
32	1.75E-11	1.93E-09	1.75E-11	0.00252
33	1.48E-12	4.96E-10	1.48E-12	0.00113

**Figure 1:**
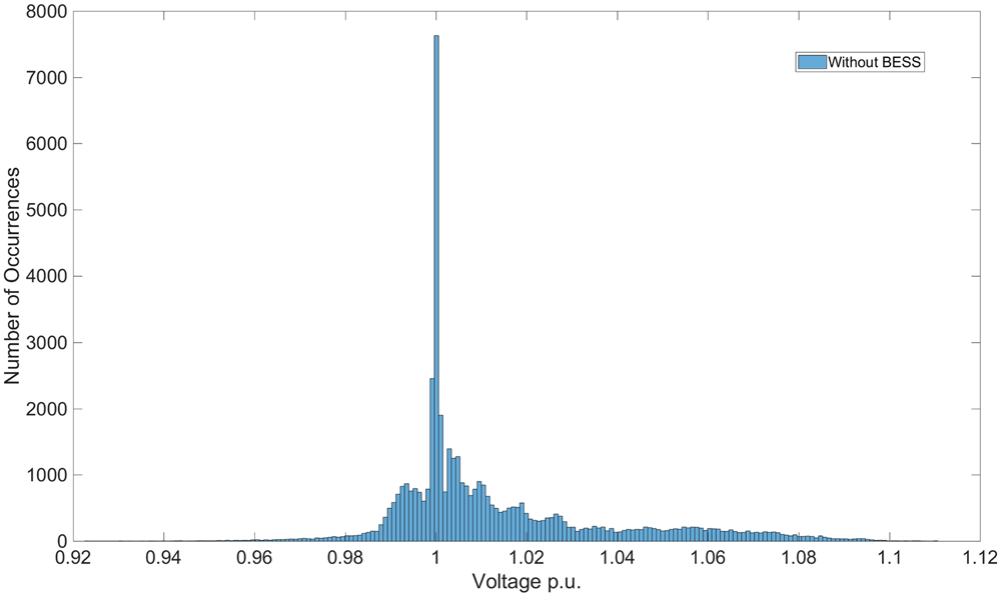
Histogram of Voltage fluctuations in case of the system without ESS.

**Figure 2:**
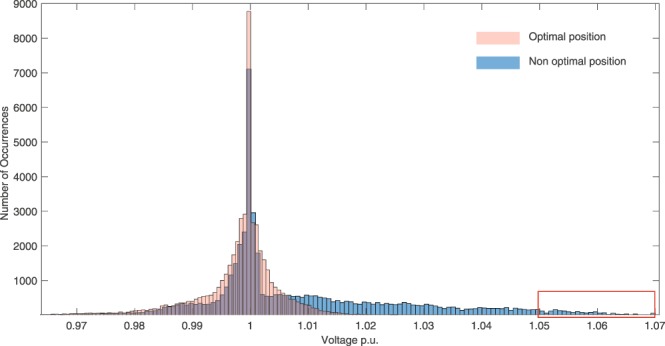
Comparison of histograms of voltage fluctuations in case of optimal (i.e. lower centrality) and non optimal (i.e. higher centrality) position of the ESS.

Considering the voltage fluctuations and Fig. 1 reports the histogram of the voltage fluctuation without ESS for the 69 IEEE network, and shows how they are distributed in the range [0.94, 1.1]. By using the ESS in the optimal position, the voltage fluctuation are found in the narrower, better range [0.97, 1.02], as showed in Fig. 2. This last figure also shows that even in non optimal positions (node 2), the ESS still positively affects the voltage quality. In fact, fluctuation are found in the interval [0.97, 1.07].

Looking more in detail at the voltage distribution we compare in Fig. 2 the histogram of the voltage distribution when the battery is placed in node 2 (higher centrality values) and in node 27 (lower centrality values). Positioning the battery in node 2 leads to a non optimal choice, since the voltage distribution spreads over the interval [0.97, 1.07]. This represents a criticality especially for those values going over 1.05, i.e. the quality upper bound usually considered by the power regulation authorities. On the other hand, when considering the ESS in node 27 we find that the position is optimal since the histogram of the voltage distribution shrinks within the interval [0.95, 1.02], confirming a significant increase of the voltage quality.

Therefore, positioning the battery in a node with low centrality (i.e. peripheral node) yields a significative reduction of fluctuations. The reliability of this assumption is also confirmed by the literature, were similar findings are reported for some specific case studies analyzed by means of OPF methods^21–23^.

**Figure 3:**
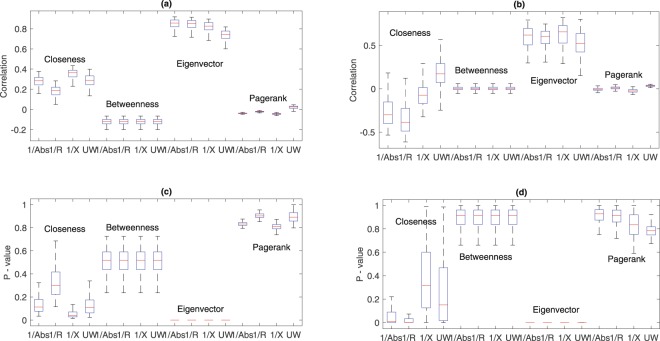
Values of correlation functions for the IEEE 33 and 69 bus. (**a**,**c**) IEEE 33 Bus correlations and *p*-values. (**b**,**d**) IEEE 69 Bus correlations and *p*-values.

Starting from the specific case of nodes 2 and 27, we consider all the nodes and the 100 configurations by performing the correlation analysis. Figure 3 shows the result of the correlation analysis performed on both the IEEE 69 and 33 bus. Referring to the IEEE 33 Bus, panel (a) reports the boxplot of the exponential correlations computed considering the 4 metrics for all the weighting models of the networks (see methods for the weight definition), while panel (c) corresponds to the *p*-values computed to validate the correlations. Results show that correlation values range from 0.7 to 0.8 in the case of Eigenvector centrality, while the other metrics show lower correlations than Eigenvector centrality. With regards to validation, we notice in panel (c) that the only significative correlation is with Eigenvector centrality, while the other do not appear to be valid under the statistical point of view. A similar result is found when analyzing the results for the IEEE 69 Bus: the correlations are weaker (~0.6) and the *p*-values are under the 10^−3^ threshold. The other centrality measures do not appear to be correlated with the quality of the voltage Δ_*q*_.

**Figure 4:**
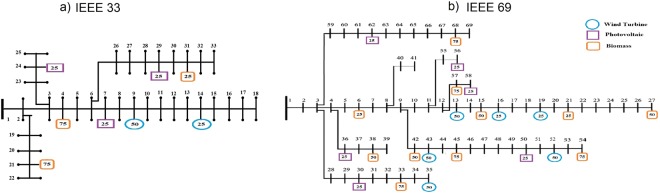
The IEEE standard networks used in this paper. (**a**) IEEE 33 Bus. (**b**) IEEE 69 Bus. For both networks the position of loads and generators refers to one of the randomly generated configurations. All power values are given in kW.

Eigenvector centrality is therefore exponentially correlated with the position of the battery that maximizes the voltage quality, and we now show the effect of the battery position on the voltage fluctuations, showing that positioning the battery in the less central node improves the voltage quality.

## Discussion

Finding a statistically significant correlation between the position of storage systems and the node centrality of a power network suggests a easy and immediate criterion for the efficient and resilient planning of power networks in presence of fluctuating RES. Since the same correlation values were found considering different weighting methods, including the unweighted case, one may consider that the optimal position of storage systems (or more generally of active and reactive power compensator) strongly depends on the topological characteristics of the network. A further indication is that when dealing with voltage fluctuations in power networks, the eigenvector centrality should be preferred among the available centrality metrics.

The findings have been shown to be independent from the exact position of loads and generators in the network. Thus our results may be valuable during the planning procedures of newly or existing power grids, including those for which a further expansion process in terms of users and distributed generation is expected. Furthermore, the reliability of our results is confirmed by the technical literature and by the common technical practice to place active and reactive power compensator near or in the end nodes to reduce voltage fluctuations. Future efforts will be devoted to include the finding of this paper in a bigger framework aimed at the passive optimization and resilience of microgrids considering a scenario of full deployed distributed energy sources and microgrids.

## Methods

Modern power grids are designed to transmit and distribute electricity from generators to end users. In order to be effective, the delivered electricity should complain to well defined physical characteristics. Regarding the Alternate Current (AC) delivered in medium and low voltage grids, the most common form of electricity delivered to end-users, voltage and power are the most important physical observables and should be kept between well defined constraints in order to ensure the proper operation of the attached electrical devices. The steady state of these two quantities is ruled by power flow equations, given in subsec. together with a quick description of the physical quantities of interest. In medium and low voltage grids, one of the main tasks of the Distribution System Operators (DSO) is to provide voltage control over the grid, i.e. to ensure that the delivered electricity is constrained between the given voltage limits. However, this task is getting more and more difficult with the increasing penetration of RES. In fact, an high share of intermittent and non-controllable power output of these energy sources can easily modify the voltage distribution in a power grid, moving the voltage levels of some nodes outside the limits and undermining the quality of the delivered electricity. This, in turn, can lead to sudden detachment of interested nodes causing a loss of supply and, ultimately to a loss of money and resources to the society and the DSO. In order to avoid this, various devices have been developed which make the DSO able to control nodes voltages. Among them, one of the most effective ones is the reactive power control, which consists in controlling the network phases *φ* in order to modify its **V** and **S** values. In this framework, ESS are very useful features for power and voltage control. In fact, the inverters used for Direct Current (DC)-AC conversion of their power output can be actively controlled, when needed, in order to provide active and reactive power to the system. In this view, ESS can be a powerful resource for DSO for ensuring the power quality over its controlled grid, and it becomes important to understand where to install these resources in order to optimize their positive impact on the system.

Our computations are based on the definition of the optimal usage of ESS for active and reactive power control. In particular, we identify the better usage scheme for an ESS from both technical and economic point of view, and to this aim Optimal Power Flow (OPF) methods are widely used in electrical engineering field for the identification of the better usage scheme of energy production facilities^24^. The aim of OPF is the optimization of the steady state variables of power grids in terms of the working parameters of the system generation facilities, under pre-defined constraints in terms of power quality. However, the classic single period optimizations are not suitable for the optimization of power systems including ESS because of the the fact that the ESS energy availability is strongly time dependent, and the past and future ESS usage cannot be decoupled from the real-time optimization. For this reason, in last years, Multi-Period OPF (MPOPF) techniques have been introduced for the optimization of ESS usage schemes, and are considered the state of the art for this type of analyses^25^. The outcome of the analysis consists in the definition of the optimal usage of the generators and ESS of the investigated systems both from economic and technical point of view. Also, this leads to the definition of the system operative parameters such as power flows, losses, and node voltages in the optimal configuration.

### Node centrality

In complex networks science, centrality is a cencept related to the importance that a given node has in the network. There are a number of characteristics, not necessarily correlated, which can be used in determining the importance of a node. These include its ability to communicate directly with other nodes, its closeness to many other nodes, and its indispensability to act as a communicator between different parts of a network. Considering each of these characteristics in turn leads to different centrality measures. For an extensive description of the centrality measures the reader is referred to^26^.

### Power flow methods

Power Flow (PF) equations describe the steady state of power systems in terms of complex quantities regarding power **S**_**i**_ and voltage **V**_**i**_ at node *i* and current **I**_**ij**_ between nodes *i* and *j*. These three quantities are related by eq. 1 where **S**, **V** and **I** are given in matrix terms. Thus, only two of these three quantities are independent, and the quantity **I**_**ij**_ is considered the dependent variable in most of the applications.

1 $${\bf{S}}={\bf{V}}{{\bf{I}}}^{\ast }$$

Generally, **S**_**i**_ = *P*_*i*_ + *iQ*_*i*_ is given in terms of its real and imaginary parts *P*_*i*_ and *Q*_*i*_, called active and reactive power respectively. Also, ${{\bf{V}}}_{{\bf{i}}}={V}_{i}{e}^{i{\theta }_{i}}$ is given in terms of its magnitude *V*_*I*_ and phase *θ*_*i*_. Expanding eq. 1, it is possible to obtain the power flow equations given in Eqs 2 and 3, where *G*_*ik*_ and *B*_*ik*_ are the real and imaginary parts of the admittance *Y*_*ik*_ = *G*_*ik*_ + *B*_*ik*_ between nodes *i* and *k*. Solution of this non-linear set of equations allows the identification of the steady state of the system. The non-linearity of the set of equations can be solved with different numerical approximation methods, a list of which can be found in^27^.

2 $${P}_{i}=\sum _{k=1}^{N}\,|{V}_{i}||{V}_{k}|({G}_{ik}\,\cos ({\theta }_{ik})+{B}_{ik}\,\sin ({\theta }_{ik}))$$

3 $${Q}_{i}=\sum _{k=1}^{N}\,|{V}_{i}||{V}_{k}|({G}_{ik}\,\sin ({\theta }_{ik})-{B}_{ik}\,\cos ({\theta }_{ik}))$$

As a general point, given two quantities between *P*_*i*_, *Q*_*i*_, *V*_*i*_ and *θ*_*ik*_ per each node and a given reference, the solving of PF equations allows the understanding of the steady state of the system. In this way, by knowing the state of each node and line in the system, it is possible to verify the adherence to constraints of the system itself. By using these equations and quantities, it is then possible to test the impact on the system given by changes in power injection on the nodes (or in applied voltage), and to identify a solution which mets the given contraints, optimizing at the same time the system with respect to a given variable. This methodology is called Optimal Power Flow (OPF), and it is often performed in order to obtain an economical optimization of the system. If this optimization is made over multiple time periods, it takes the name of Multi-Period Optimal Power Flow (MPOPF). This methodology is extremely useful when dealing with storage systems, for which the amount of power which can be injected at a certain time strongly depends on their state of charge, and thus on the usage made in the previous time frames.

To compute the voltage distributions we used a MPOPF method based on Genetic Algorithms optimization (GA-MPOPF), first proposed in^9^, and now improved with a further reactive power optimization step, described in detail in the following. This method has been used for the estimation of the varying impact of ESS on voltage regulation, when positioned on different nodes of the system.

The results of the GA-MPOPF method have then been analyzed from a complex networks perspective, by studying the correlation between the obtained results and the centrality of the nodes on which the ESS has been placed. Indeed, centrality is a well-known concept in complex networks theory, and it is based on the definition of measures for the quantification of the importance of nodes (or edges) into a given graph. Since the importance of the nodes in a graph is dependent on the type of network they belong, different centrality measures have been proposed in the past. Among them, the most important centrality measures are the betweenness centrality, the eigenvector centrality and the closeness centrality. These measures have been proven to be meaningful in a great variety of networks, covering different fields like sociology, economics and finance, physics^26^. Also, they have been shown to be important measures of importance in various applications of complex networks theory to power grids^4^, especially when their electric counterparts, defined on the base of electric distances, are considered.

**Figure 5:**
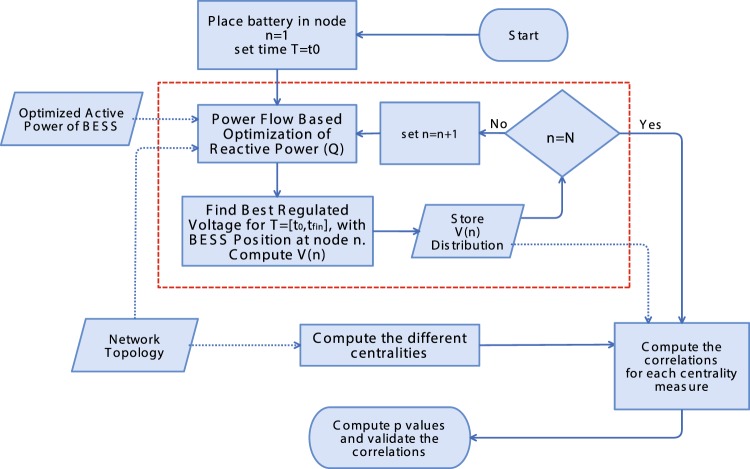
Flowchart describing how the computation procedure described in this paper works, including the computation of inter-quantile differences Δ_*q*_ and the centrality metrics The improved GA-MPOPF method is enclosed in the red box.

The flowchart of Fig. 5 describes the process that we used to compute the centrality metrics and the voltage distributions *V*(*n*). The flowchart is composed by two main parts. The first one, describes how the centrality metrics are computed starting from the power network topology: we transform the network into a weighted graph, whose nodes (1, …, *N*) represent the loads and generators, while the edges represent the electrical connections between two nodes *i* and *j*, weighted according to the following models:

1. Inverse of reactance (susceptance) weight: *w*_*ij*_ = 1/*X*_*ij*_
2. Inverse of resistence (conductance) weight: *w*_*ij*_ = 1/*R*_*ij*_
3. Absolute value of the inverse of impedance (admittance) weight: *w*_*ij*_ = 1/||*R*_*ij*_ + *jX*_*ij*_||
4. No weight: *w*_*ij*_ = 1 if *i*, *j* are connected, otherwise *W*_*ij*_ = 0.

Considering these weights we compute for each node the following centrality metrics: (1) Closeness, (2) Betweenness, (3) Eigenvector, (4) Pagerank.

The second part of the flowchart, enclosed in the red box, computes the voltage distribution *V*(*n*), i.e. the distribution of all the voltage values recorded in each node (1, …, *N*) between time *t*_0_ and *t*_*fin*_ when the battery is placed in node *n* considering a sampling time of 1 hour. The distribution *V*(*n*) is a direct measure of the overall quality of voltage in the power network. Here we consider that the quality of *V*(*n*) increases as the distribution around 1.0 becomes more narrow, while the range *V* ∈ (0.95, 1.05) is considered as lower bound for an acceptable quality. The procedure starts with a power flow optimization of the reactive power *Q* to compute the optimal battery capacity Q^*^(*t*). From equation 4 we compute the available reactive power *Q*.

4 $$Q(t)=\pm \,\sqrt{{S}^{2}-{P}^{2}(t)}$$

where *S* is the battery rated power, *P*(*t*) is optimal active power of battery and *Q*(*t*) is available reactive power of battery for each time interval *t*. Then for each time *t* = [*t*_0_, …, *t*_*end*_] and each node *n* = [1, … *N*] we compute the matrix of the voltage distribution:

5 $$V=(\begin{array}{ccc}{v}_{1}({t}_{0}) & \cdots  & {v}_{1}({t}_{end})\\ \vdots  & \ddots  & \vdots \\ {v}_{N}({t}_{0}) & \cdots  & {v}_{N}({t}_{end})\end{array})$$

The optimization procedure aimed at obtaining the Q^*^, which is the optimal reactive power provided by the battery and used to regulate the voltage. The value of Q^*^ has been optimized on the base of the hierarchical rules defined in Eq. 6, where *V*_*min*_ and *V*_*max*_ are the voltage limits proper of the analyzed grid, #() is the counting operator and *σ*() computes the experimental standard deviation of the sample. The *min* values have been computed on the full time interval *t*_0_ ≤ *t* ≤ *t*_*end*_.

6 $$\begin{array}{l}1)\,{\rm{\min }}\,\#(V > {V}_{max}||V < {V}_{min})\\ 2)\,{\rm{\min }}\,\#({\rm{\max }}\,\mathrm{(|1}-V|))\\ 3)\,{\rm{\min }}(\sigma \mathrm{(|1}-V|))\end{array}$$

If we compute ${Q}_{p}^{\ast }$ for each position of ESS for all times and all nodes by following the rules given in Eq. 6 we obtain the matrix:

7 $${Q}_{p}^{\ast }=(\begin{array}{ccc}{Q}_{1}^{p}({t}_{0}) & \cdots  & {Q}_{1}^{p}({t}_{end})\\ \vdots  & \ddots  & \vdots \\ {Q}_{N}^{p}({t}_{0}) & \cdots  & {Q}_{N}^{p}({t}_{end})\end{array})$$

Then, for each battery position we can compute a set of n matrices *V*^*p*^:

8 $${V}^{p}=(\begin{array}{ccc}{v}_{1}^{p}({t}_{0}) & \cdots  & {v}_{1}^{p}({t}_{end})\\ \vdots  & \ddots  & \vdots \\ {v}_{N}^{p}({t}_{0}) & \cdots  & {v}_{N}^{p}({t}_{end})\end{array})$$

where *p* = 1, …, *N* are the position of the batteries, and ${v}_{i}^{p}({t}_{k})=f({Q}_{p}^{\ast }({t}_{k}))$

The correlation is computed starting from the vector of the differences between the 75-th and 25-th quantiles of the voltage distribution *V*(*n*) (computed for each position of the battery) ${{\rm{\Delta }}}_{q}^{n}=({{\rm{\Delta }}}_{q}^{1},\,\mathrm{...,}\,{{\rm{\Delta }}}_{q}^{N})$ and the vector of the centralities of each node ${C}_{\ast }^{n}=({C}_{\ast }^{1}\mathrm{,...,}{C}_{\ast }^{n})$ evaluated for the selected metrics * = *C*, *B*, *E*, *P* (Closeness, Betweenness, Eigenvector, Pagerank). Correlations are validated by computing the *p*-value and rejecting all the correlations with *p*-values above 10^−3^.

The IEEE standard power networks used in this paper are showed in Fig. 4, where panel (a) refers to the 33 Bus and panell (b) refers to the 69 bus. Such networks are considered as standard for testing purposes by the IEEE technical literature, and a full description can be found in^18,20^. In this paper we use production and consumption data according to^8^, where real yearly profiles for load and DG are used. The studied Li-ion ESS has a capacity of 300 *kWh* and a rated power of 300 *kW* and it is used for both 33 and 69 IEEE standard networks and their different configurations.
